# pH Adaptation stabilizes bacterial communities

**DOI:** 10.1038/s44185-024-00063-5

**Published:** 2024-10-17

**Authors:** Akihiko Mougi

**Affiliations:** Institute of Agricultural and Life Sciences, Academic Assembly, Shimane University, 1060 Nishikawatsu-cho, Matsue, 690–8504 Japan

**Keywords:** Ecology, Microbiology

## Abstract

Diverse microbes in nature play an important role in ecosystem functioning and human health. Nevertheless, it remains unclear how microbial communities are maintained. This study proposes that evolutionary changes in the pH niche of bacteria can promote bacterial coexistence. Bacteria modify the pH environment and also react to it. The optimal environmental pH level for a given species or pH niche can adaptively change in response to the changes in environmental pH caused by the bacteria themselves. Theory shows that the evolutionary changes in the pH niche can stabilize otherwise unstable large bacterial communities, particularly when the evolution occurs rapidly and diverse bacteria modifying pH in different directions coexist in balance. The stabilization is sufficiently strong to mitigate the inherent instability of system complexity with many species and interactions. This model can show a relationship between pH and diversity in natural bacterial systems.

## Introduction

How diverse organisms coexist in natural ecosystems remains a longstanding puzzle in ecology. Ecological theory suggests that communities with numerous interacting species are inherently unstable^1^, posing a challenge to the maintenance of species diversity. While efforts to address this “diversity–stability” problem have primarily focused on macro-organisms such as animals and plants^2,3^, microbial communities—comprising a vast array of species essential to ecosystem function^4–8^—present a unique yet underexplored avenue for understanding stability mechanisms^9–12^.

Microbial communities exhibit distinctive features that differentiate them from macro-organisms. One notable feature is the feedback loops between their metabolic activities and environmental conditions^13–16^. This interaction is particularly evident in the modulation of pH, a crucial environmental factor influencing microbial physiology and community dynamics^17–22^. Some bacteria actively modify their surrounding pH through metabolic processes^17,23,24^, creating conditions that influence the growth and survival of coexisting species. At the same time, because pH is a key niche for bacteria, interspecific competition can occur if competing bacteria prefer a similar pH environment^25,26^.

Another distinctive feature of microbial communities is their rapid adaptation rates^15^. Microbes have generation cycles much faster than those of most animals and plants, enabling them to swiftly evolve in response to environmental fluctuations^27–29^. This rapid evolutionary adaptation plays a pivotal role in shaping ecological population dynamics and promoting species coexistence^30–35^. Bacterial species can adapt their pH preferences, or “pH niches,” in response to environmental changes^36–40^, thereby maintaining ecological balance despite potential fitness costs^41^.

Considering these two major features in bacteria—the feedback between environmental conditions and metabolic activities, and their rapid evolutionary adaptation—a hypothesis emerges: The dynamics of bacterial communities are driven by the changes in pH environment caused by themselves and their evolutionary adaptation in response to these pH changes.

**Fig. 1:**
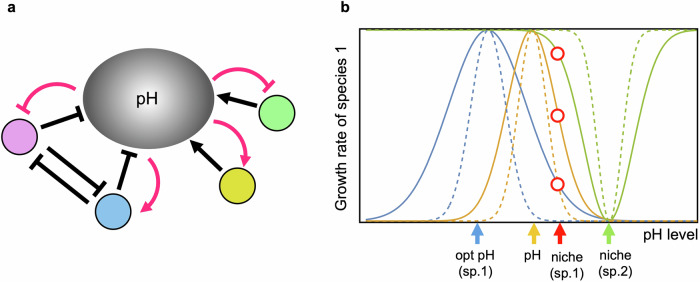
Schematic illustrations of the model. **a** Ecological relationships between bacterial species and pH. The circles, excluding the one representing pH, denote different bacterial species. Typical arrows indicate promotion, while hammerhead arrows indicate inhibition. Black arrows represent effects due to bacteria, and red arrows represent effects due to pH. Acidophilic bacteria lower pH (inhibit), while alkaliphilic bacteria increase pH (promote). pH impacts bacterial growth either positively or negatively, depending on the relationship between the current pH value and the bacterial pH niches. Even if bacterial species have similar pH niches, they may either compete for resources (depicted between pink and blue) or not. **b** Effects of key factors on the fitness of a bacterial species. The blue, orange, and green curves illustrate the cost, pH, and competition functions, respectively. Note that the cost and pH functions are positive, while the competition function is negative. The dashed lines in each color (blue, orange, green) represent higher cost, increased pH sensitivity, and narrower niches, respectively. Blue, orange, red, and green arrows indicate the physiological optimal pH, the environmental pH value, the niche position of the focal species (sp. 1), and the niche position of a competitor (sp. 2), respectively. The three red circles, from bottom to top, represent the effects of cost, pH, and competition on the fitness of species 1 at its pH niche. Bacteria can shift their pH niche if a mutant with a slightly different pH preference shows higher fitness compared to the wild type (“Methods”).

In this study, I delve into the eco-evolutionary dynamics of bacterial communities, focusing on two distinct groups of bacteria based on their different abilities to change pH (Fig. 1). Alkaliphilic bacteria, such as *Clostridium perfringens*, *Corynebacterium ammoniagene*, and *Pseudomonas veronii*, increase pH levels, while acidophilic bacteria, such as *Bifidobacterium*, *Lactobacillus plantarum*, and *Serratia marcescens*, decrease pH levels^42–44^. The interaction between these two types of groups, mediated by their pH-modifying capabilities and adaptive responses, forms the core of the investigation into bacterial community stability (“Methods”). The goal of this study is to reveal how the pH niche adaptation in response to pH change caused by themselves affects to the stability of complex microbial communities with alkaliphilic and acidophilic bacteria. The stability is evaluated by community persistence which is the probability that all species persist for a given time (“Methods”). By elucidating how pH niche adaptation influences the persistence and dynamics of complex bacterial communities, this study aims to contribute to our understanding of fundamental ecological principles governing microbial ecosystems. Through a theoretical approaches, I explore the mechanisms underlying community stability, providing insights into how bacterial communities maintain biodiversity and ecosystem function in diverse environments.

## Results

**Fig. 2:**
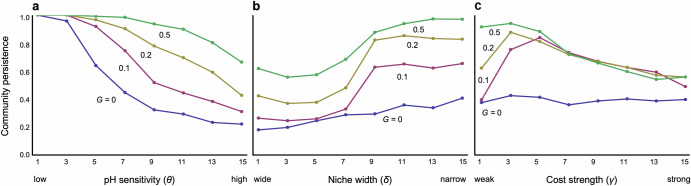
Effects of speed of adaptation on community persistence with various levels of key parameters. **a** Effects of pH sensitivity. **b** Effects of niche width. **c** Effects of cost constraint. Different colors represent different levels of speed of adaptation G. Parameters in each panel are: **a** *δ* = 15, *γ* = 2; **b** *θ* = 8, *γ* = 2; and **c** *θ* = 8, *δ* = 15. Other parameter values are *N* = 30, *C* = 0.2, *q* = 0.6, and *m* = 0.02.

Consider an extreme case without an evolutionary adaptation in pH niches. In this scenario, if pH has a minimal effect on bacterial growth rates (low *θ*), the bacterial community tends to remain stable (Fig. 2a). However, when pH significantly affects growth rate (high *θ*), the stability of the bacterial community diminishes (Fig. 2a). This dynamics also applies to in interspecific competition resulting from overlapping pH niches between species. Coexistence is less likely in the systems with wider niche widths (low *δ*) than narrower ones (high *δ*) (Fig. 2b). The effects of these two parameters on stability are consistently valid across both parameter spaces (Fig. S1a). These outcomes stem naturally because species cannot adapt to changing the pH, even if changes in environmental pH or significant overlap in pH niches between species greatly reduce growth rates.

**Fig. 3:**
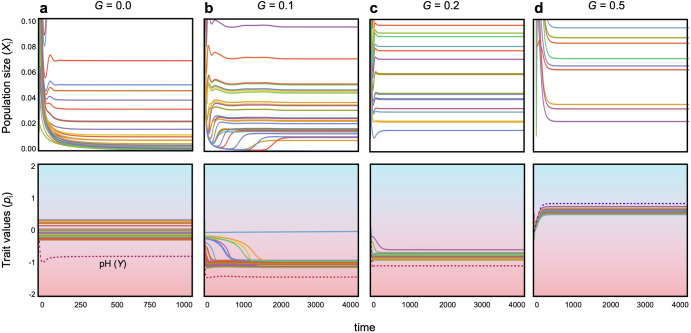
Examples of dynamics with and without evolution. The upper and lower panels illustrate population dynamics and evolutionary dynamics, respectively, across different adaptation speeds. **a** *G* = 0; **b** *G* = 0.1; **c** *G* = 0.2; **d** *G* = 0.5. In each panel, lines of different colors represent different species. In the lower panels, the dotted lines show the dynamics of pH (*Y*). The contours in the lower panels represent pH values. Parameters are: *N* = 30, *C* = 0.2, *q* = 0.6, *m* = 0.02, *θ* = 5, *δ* = 5 and *γ* = 2.

However, once pH niches evolve, each bacterial species can adapt to changes in environmental pH, fostering the coexistence of diverse bacteria species (Figs. 2a, b and 3). Community dynamics tend to stabilize further when adaptation occurs more rapidly (Figs. 2a, b and 3) and when the cost of trait changes is moderate (Fig. 2c). However, overly low costs can impede stabilization, particularly when adaptation rates are slow (Fig. 2c). Stronger costs appear to hinder adaptive changes, resulting in destabilization, while weaker costs facilitate adaptation but may lead to competitive exclusion of less fit species because superior species can easily maximize the growth rates. Hence, faster and less costly evolution of pH niches plays a pivotal role in stabilizing bacterial community dynamics. The effects of *θ* and *δ* on stability are consistently valid across both parameter spaces, while higher pH sensitivity can still lead to some degree of stabilization even with a wider niche width, contrary to the case without evolution (Fig. S1b). A possible reason is the following: If the niche width is too broad, the cost of avoiding competition becomes prohibitive, causing all bacteria to be equally affected by competition, and survival differences are likely to arise mainly based on their ability to adapt to pH environments. On the other hand, when the niche width is narrower, bacteria that can effectively balance avoiding competition and adapting to pH changes are likely to have an advantage. This can lead to significant differences in survival relative to inferior species and make coexistence more difficult.

**Fig. 4:**
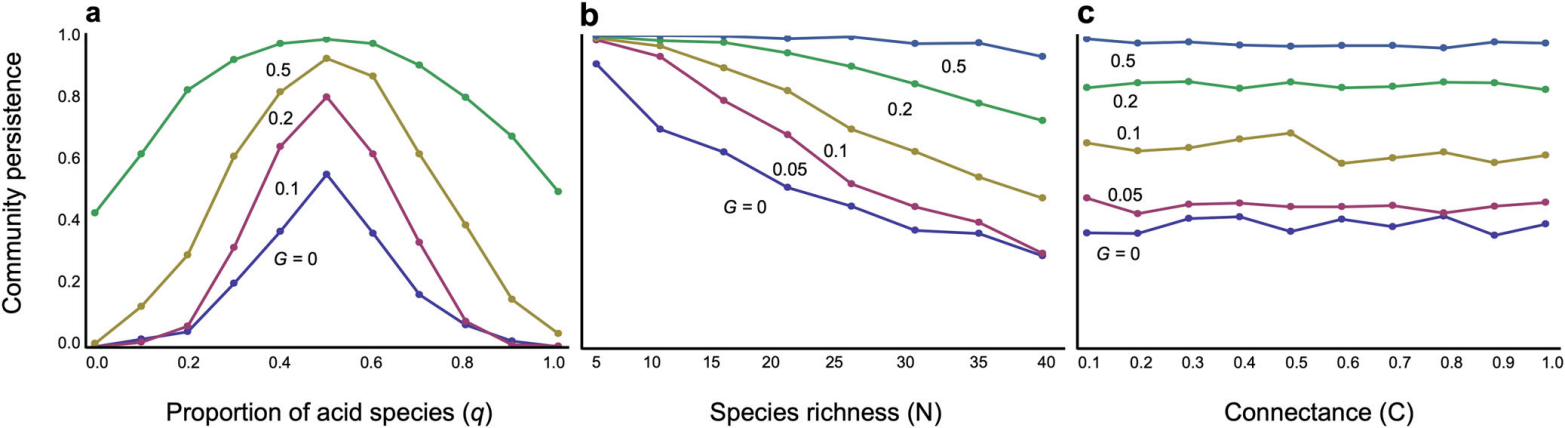
Effects of speed of adaptation on community persistence with varying community structures. **a** Community composition (the proportion of acidophilic species, *q*) is changed. *N* = 30 and *C* = 0.2 are assumed. **b** Species richness is changed. *C* = 0.2 and *q* = 0.6 are assumed. **c** Connectance is changed. *N* = 30 and *q* = 0.6 are assumed. Other parameter values are *m* = 0.02, *θ* = 8, *δ* = 15, and *γ* = 2.

The composition of bacterial species within a community significantly influences community stability. As anticipated, a balanced mix of alkaliphilic (pH-increasing) and acidophilic (pH-decreasing) species maximizes stability (Fig. 4a). Any imbalance, where one type of bacteria predominates, can markedly reduce community stability (Fig. 4a). Nonetheless, faster adaptation can mitigate destabilization stemming from an imbalanced composition, promoting relatively high stability across a broad range of community compositions (Fig. 4a).

**Fig. 5:**
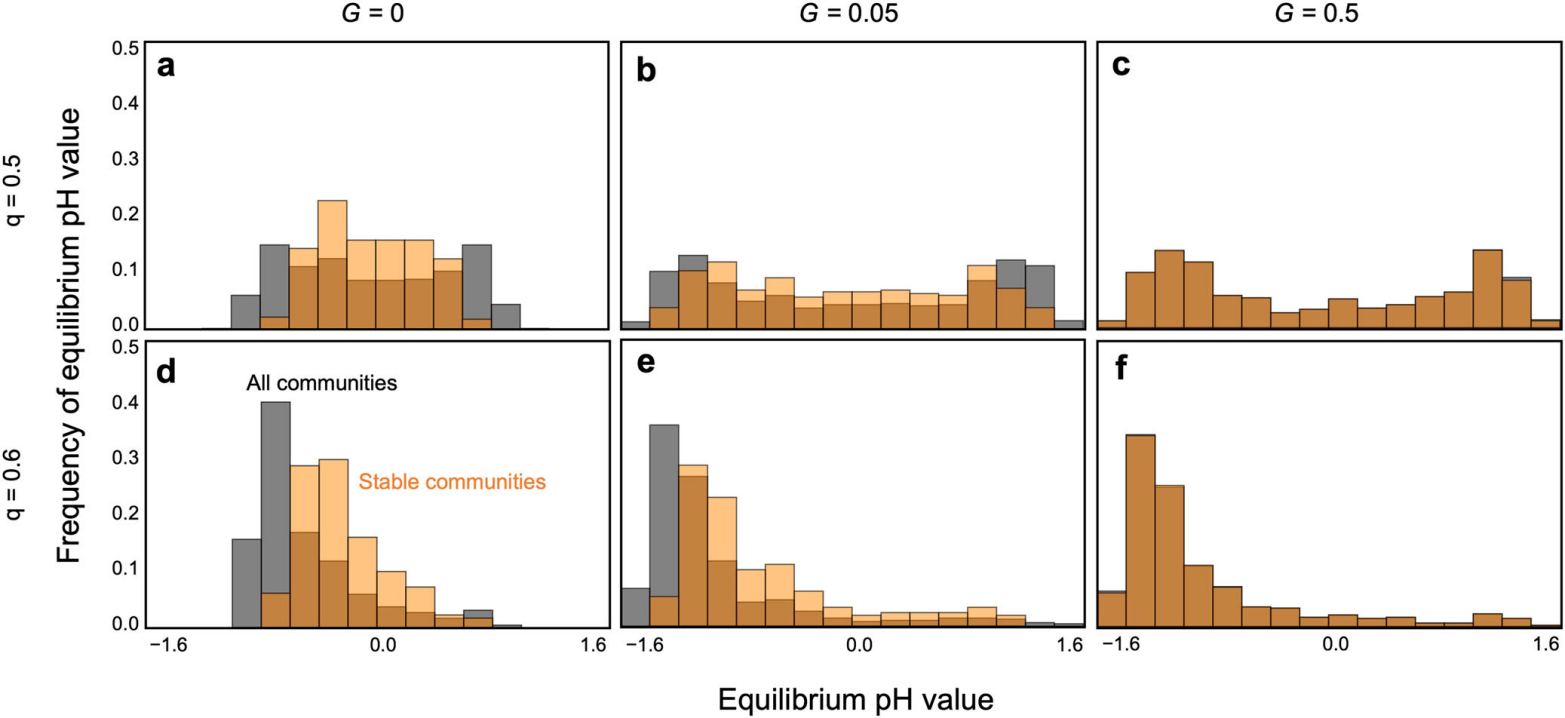
Effects of community composition and evolution on the equilibrium pH value. **a**–**c** Perfect balanced community composition (*q* = 0.5). **d**–**f** An imbalanced community composition (*q* = 0.6). **a**, **d** Without evolution (*G* = 0). **b**, **e** Slow adaptation (*G* = 0.05). **c**, **f** Fast adaptation (*G* = 0.5). Gray bars are the histograms of equilibrium pH value. Orange bars represent histograms of equilibrium pH value in only persistent communities. The dark orange regions are resultants of overlapping gray and orange bars. In (**c**, **f**), gray and orange are almost overlapped because the community persistence is very high. Parameter values are *N* = 30, *C* = 0.2, *m* = 0.02, *θ* = 8, *δ* = 15, and *γ* = 2.

It is evident that communities tend to be more stable at nearly neutral pH in the absence of evolution (Fig. 5a, d). This conclusion is supported by a mathematical analysis of a two-species system (Supplementary Information), which predicts that stable systems with balanced pH require species that prefer pH values opposite to those they produce. However, once evolution occurs, pH values can shift towards more alkaline or acidic conditions. Even within a balanced community composition (*q*$\,\approx \,$0.5), pH tends to lean towards either alkaline or acidic extremes. Evolving communities can persist even in such biased pH environments, especially with rapid evolution (Fig. 5b, c). This bimodal pH equilibrium can persist unless both pH sensitivity and cost constraints are severe (Fig. S2). In contrast, imbalanced community compositions tend to result in pH extremes depending on the predominant species. For instance, if acidophilic species dominate, the environment is likely to become acidic (Fig. 5d–f). Such extreme pH environments tend to destabilize communities, particularly when adaptation rates are slow (Fig. 5e).

Stable and unstable communities at evolutionary steady states exhibit distinct characteristics. Stable communities converge towards similar pH niches (Fig. S3), suggesting influence from the dominant species on environmental pH changes. For example, communities with more acidophilic species tend towards acidic niches (Figs. S4, S5). In contrast, unstable communities display wider variations in pH niches among species (Fig. S3). This variability arises because unstable communities often evolve towards divergent pH niches, potentially at extreme ends of the pH spectrum. For example, surviving species may prefer acidic environments while extinct species may have adapted to alkaline environments (Fig. S6).

The evolution of the pH niche also has profound implications for the complexity–stability debate. Without evolutionary changes in pH niches, increased community complexity tends to decrease stability, as expected from an earlier theory^1^. Greater species richness generally reduces stability (Fig. 4b), whereas an increase in connected competitors does not necessarily destabilize communities (Fig. 4c). This trend towards destabilization with increased species richness suggests that larger bacterial systems may struggle to persist without evolutionary adaptation of pH niches. However, evolutionary changes in pH niches can mitigate this destabilization, particularly with faster adaptation rates (Fig. 4b, c). Stability can also increase when^1^ pH self-regulation is stronger (Fig. S7a)^2^, interspecific competition is weaker (Fig. S7b)^3^, the rate of pH change is lower (Fig. S7c), and^4^ physiologically optimal pH values are closer to neutral (Fig. S8).

## Discussion

Evolutionary changes in pH niches play a crucial role in maintaining communities with both alkaliphilic and acidophilic bacteria, especially when adaptation occurs rapidly. Faster adaptation helps uphold these communities, except when there is a significant imbalance in the composition or ratio of alkaliphilic to acidophilic bacteria. Communities tend to persist when species converge to adapt to the pH environment influenced by the majority of bacteria types. Conversely, divergent evolution towards vastly different pH niches undermines community persistence. The stabilizing effect of rapid evolution effectively mitigates the inherent destabilization associated with increased community complexity or species diversity. These findings underscore the self-sustaining nature of microbial communities with alkaliphilic and acidophilic bacteria, adapting to pH changes induced by the community itself.

Divergent or convergent evolution in pH niches is influenced by the predominant bacterial types shaping the pH environment. When a majority of bacteria create a pH environment biased towards one direction, there is a tendency for other species to adapt their pH niches to match the majority. This matching can be advantageous initially, as it allows species to thrive in the prevailing pH conditions. However, it also intensifies interspecific competition by creating niche overlaps among bacterial species. Species that are weaker or have a suboptimal pH niche, characterized by a low growth rate or an optimal pH value distant from the majority, face challenges in surviving. If these species cannot adapt quickly enough to the pH environment set by the majority, they may fail to persist (Fig. S9). Conversely, even if they adapt to match the majority, they may still struggle due to strong competition from numerous competitors. In such a dilemma, species with faster adaptation rates can simultaneously match their pH niche to the majority and partition their niche space, even among weaker or less well-adapted species. This process promotes convergent evolution towards similar pH niches within the community. On the other hand, slow adaptation rates (noting that evolution in weaker or less well-adapted species progresses slower due to smaller fitness gradients) hinder species from effectively matching the pH niche of the majority and maintaining distance from majority pH niches. Slow adaptation makes it challenging to finely tune pH niches to avoid niche overlaps. Even with significant niche partitioning from the majority, weaker or less well-adapted bacteria struggle to survive in an unfavorable pH environment. This underscores the critical role of adaptation speed in determining whether species converge towards similar pH niches or maintain distinct, potentially divergent niches.

Empirical works have shown that soil pH is a major driver of the soil communities^40,45–49^. The soil pH significantly explains the community composition and diversity of soil bacterial communities. Soil communities can show different patterns in the relationship between pH and diversity. In addition to peaking around nearly neutral pH^50,51^, soil communities shows an increasing community composition and diversity with an increase in pH^45^. The theory proposed here suggests that communities tend to be stable under nearly neutral pH conditions, particularly when the community composition is balanced between alkaline-producing and acid-producing bacteria, and there is high pH sensitivity and a substantial cost associated with trait changes (Fig. S2). Therefore, such conditions are crucial for the peak diversity observed around nearly neutral pH. Furthermore, this theory posits that when the community composition is biased towards one type of bacteria, the peak diversity shifts towards a particular pH direction (Fig. 5, Fig. S2). Such biases may lead to another pattern where pH increases are associated with an increasing bias towards one bacterial type. The present study also predicts that under conditions of nearly balanced community composition and moderate pH sensitivity, two peaks of diversity can emerge (Fig. S2). Considering these complex interrelationships and feedback loops, the question arises: what drives the relationship between pH and diversity? The present study suggests that adaptation to pH changes plays a pivotal role in maintaining species diversity. Indeed, empirical evidence has shown that bacterial species capable of acid tolerance thrive in strongly acidic environments, providing them with a competitive advantage over other bacteria^45^. The ability of species to persist or decline is largely determined by their speed of adaptation to environmental changes.

pH adaptation could play a key role in bacterial community dynamics. In a healthy state, the dental community hosts numerous bacteria capable of alkalinizing acids and maintaining health by neutralizing them. However, when there is a disturbance (resulting in a decrease in pH), acid-producing bacteria and those adapted to acidic conditions form a community that excludes others, creating a lower pH environment and preventing dental caries^52,53^. Such adaptations to pH changes in communities, along with alterations in their composition, are also observed in bacteria found in wastewater^54^, soil^55^, and coral reefs^56,57^. Moreover, studies investigating the stability of microbial communities in response to pH disturbances have indicated that microbial diversity is linked to stability^58^. However, in the present study, it was found that species diversity alone does not contribute to stability unless the rate of adaptation is rapid. Therefore, research that tracks adaptive changes^59^ is crucial to test this theoretical prediction.

It is possible to test this theory in both experimental and field studies. In a simple experimental study with various types of bacteria species on the metabolic properties of pH, each bacteria species largely changes the pH value by themselves in the environment. In addition, it also largely affects to the population dynamics, resulting in coexistence or competitive exclusion. However, the experiment is based on the ecological dynamics in which evolution does not occur^12,44^. Hence, if we can control the evolution of traits on pH niche (such as preference or tolerance) and trace the trait dynamics in diverse bacteria species, it can be made close to the present model situation. If it is possible, the destabilization effect of strong interactions among bacteria species (high value of pH change rates) shown by an experimental study^12^ may be strongly weakened by rapid pH adaptation. On the other hand, in the field study, the situation is more complicated. In addition to a similar examination with the lab experiment, it needs to examine what causes pH change and how much the microbes affect to the pH dynamics in the broad environment. It also needs to know the metabolic properties of pH in each diverse species. A nonlinear time series analysis^60^ can help to capture the effects of each species on pH dynamics.

The model assumed a particular type of microbe. Bacterial growth tends to be favored within optimal growing conditions, and bacteria are capable of altering their surrounding pH. However, in nature, there are diverse types of microbes. For instance, under stressful environments, microbes can accelerate their growth rates^61^. Some microbes possess mechanisms to adapt to pH changes without altering their pH niche^41^. Furthermore, various types of interactions are present in microbial communities^62^. Understanding and integrating these diverse microbial characteristics and interactions for community stability will be a major goal in future studies.

The present study shows that the speed of evolution and community composition (alkaline producing vs acid producing) play a key role in maintaining large bacterial communities. The speed of evolution is related to genetic diversity, and the community composition is related to the diversity of metabolic properties among species. The present findings suggest that species diversity is maintained by genetic diversity and metabolic diversity.

## Methods

### Ecological community dynamics model

Consider a bacterial community in which each bacteria species indirectly interacts through the pH changes caused by each species. One type of bacteria increases pH (alkaliphilic bacteria), whereas the other type decreases pH (acidophilic bacteria). Because pH is a key parameter that effects the bacteria growth rates, a pH that deviates from their preferred pH niche decreases their growth rates. Bacteria also compete if they share a common niche. Here the niche is assumed to be strongly related to their preferred pH environment, which implies that, as their preferred pH becomes similar, the preferred habitats become similar and they are more likely to share common resources. The pairs of bacteria species *i* and *j* (*i*, *j* = 1, …, *N*) are connected by a competitive interaction with a probability of *C*, which is defined as the proportion of realized interaction links *L* in the possible maximum interaction links *L*_*max*_ of a given network model (*L = CL*_*max*_). The competitive interaction network was randomly constructed. The dynamics of population sizes of each bacterial species and pH levels in this scenario are described by the following differential equations:

1 $${{dX}}_{i}/{dt}=\left({r}_{i}-{X}_{i}-{\sum }_{j}{a}_{{ij}}{X}_{i}\right){X}_{i}$$

2 $${dY}/{dt}={\sum }_{j}{k}_{j}{X}_{j}-{mY}$$

where *X*_*i*_ is the abundance of species *i*; *r*_*i*_ is the intrinsic growth rate of change in species *i; α*_*ij*_ is the competition coefficient, defined as the relative strength of interspecific competition to intraspecific competition; *k*_*i*_ is the rate of pH change caused by each bacteria (*k*_*i*_ < 0 in acidophilic bacteria, whereas *k*_*i*_ > 0 in alkaliphilic bacteria); and *m* is a self-regulation parameter to avoid extreme pH values. For example, plants or minerals buffer the pH in soils around them so as to avoid extreme pH values^63,64^. The proportion of acidophilic bacteria within a community is controlled by *q* (i.e., the proportion of alkaliphilic bacteria is 1–*q*).

The growth rate of each bacteria is maximized at a preferred pH level, *p*_*i*_. If the preferred pH is a physiologically optimal value ${\bar{p}}_{i}$ (*p*_*i*_ = ${\bar{p}}_{i}$), the growth rate is described by an inverse bell-shaped function (*r*_*i*_ = ${{r}}_{0i}{e}^{{-\theta \left(Y-{p}_{i}\right)}^{2}}$) where *r*_0*i*_ is the maximum growth rate for each bacteria species and *θ* (>0) is the shape parameter of the function (pH sensitivity). As *θ* increases, the steepness of the function increases, implying that pH preference is narrow. The pH preference is clearly crucial to bacterial fitness, which strongly suggests that pH preference is a key selection target trait that will evolve in response to changes in environmental pH. To consider the evolution of pH preference in bacteria, the cost constraint for changing pH preference is assumed. Because a deviation from the physiologically optimal trait value is assumed to decrease the growth rate, the growth rate with a specific cost function is given by *r*_*i*_ = *r*_0*i*_${e}^{{-\theta \left(Y-{p}_{i}\right)}^{2}}{e}^{-{\gamma \left({\bar{p}}_{i}-{p}_{i}\right)}^{2}}$, where *γ* represents the cost strength. A large value of *γ* implies that the trait change is very costly.

In this study, pH is a key niche and an overlap of pH preferences among bacteria species can imply an overlap of spatial and/or food resources. Thus, interspecific competition can increase as the pH preferences of each bacteria species become more similar. A specific competition function is given by: *α*_*ij*_ = *α*_0*ij*_$\scriptstyle{e}^{{-\delta \left({p}_{i}-{p}_{j}\right)}^{2}}$, where *α*_0*ij*_ is the maximum competition coefficient in each bacteria species (the effect of species *j* on *i*) and *δ* (> 0) is the shape parameter of the function that reflects the niche breadth. A large value of *δ* implies that the niche breadth is very narrow, whereas a slight deviation between their trait values can greatly weaken the interspecific competition. On the other hand, sharing a pH niche does not necessarily mean sharing a resource niche. This is reasonable in cases where multiple types of resources exist within a similar pH environment. In such case, even if species prefer similar pH conditions, they do not necessarily share the same resources. The presence or absence of competition is determined by *C*.

### Evolutionary dynamics

The adaptive dynamics of the mean trait value (*p*_*i*_) are modeled using a quantitative trait evolution model^65^:

3 $$\frac{d{p}_{i}}{{dt}}={G}_{i}{\left.\frac{\partial {W}_{i}}{\partial {p}_{{\rm{m}}i}}\right|}_{{p}_{{\rm{m}}i}={p}_{i}}$$

where *G*_*i*_ (*i* ∈ 1, 2) represents a control parameter that represents the speed of adaptation^65^. The value *W*_*i*_ is the fitness of the mutant trait (*p*_m*i*_), which is defined as the per capita growth rate of the mutants: *W*_*i*_ *= r*_*i*_ *(p*_*mi*_*)-X*_*i*_ *- α*_*ij*_ *(p*_*mi*_*,p*_*j*_*)X*_*j*_. Equation (3) indicates that the rate of adaptive change in the traits should be proportional to the selection gradient ($\scriptstyle{\left.\frac{\partial {W}_{i}}{\partial {p}_{{\rm{m}}i}}\right|}_{{p}_{{\rm{m}}i}={p}_{i}}$). If the selection gradient is positive, then the selection pushes the population toward higher trait values; in contrast, if it is negative, then the selection pushes the population toward lower trait values. At evolutionary equilibrium, the selection gradient becomes zero.

### Eco-evolutionary dynamics

The differential equations of coupled population and trait dynamics (Eqs. (1)–(3) were calculated using a computer simulation, and the effects of trait dynamics on population dynamics were examined. When a sufficiently long simulation run (t = 2 × 10^5^) was used to evaluate system stability in the equilibrium state, asymptotic behavior was obtained (the last 10^3^ steps were used to calculate stability index as defined below). Iterated simulation runs were performed with randomly chosen parameter sets (determined from a uniform distribution with each parameter range): *r*_*0i*_ = 0.1–1.0; $\left|{k}_{i}\right|=0-0.1$; and *α*_0*ij*_ = 0–0.1 (larger maximum values of $\left|{k}_{i}\right|$ and *α*_0*ij*_ were tested in Fig. S7). Here, the biologically feasible assumption was that interaction strengths decrease with increasing interaction pairs because of an allocation of interaction effort. To simply consider this assumption, the actual values of *α*_0*ij*_ are divided by the number of competitors.

The initial values of abundance, pH level, and traits were also randomly set in each simulation run. The initial values of species abundance and pH level were randomly selected from a uniform distribution: *X*_*i*_ = 10^−2^ to 10^−1^ and *Y* = −10^−3^ to 10^−3^, respectively. The initial values of *p*_*i*_ (= ${\bar{p}}_{i}$) were randomly selected from a normal distribution with mean = 0 and sd (σ) = 0.2 (other values of σ were tested in Fig. S8). Community persistence, which is used as a representative of stability, was calculated by measuring the fraction of simulation runs in which the entire community persisted (*X*_*i*_ > 10^−4^ for all *i*) after a sufficiently long period (see above) of 500 runs.

## Supplementary information

Supplementary Information

## Data Availability

No datasets were generated or analysed during the current study.
