# Supplementary material for: pH Adaptation stabilizes bacterial communities
Source: NPJ Biodivers. 2024 Oct 17;3:32. doi: 10.1038/s44185-024-00063-5 (PMC11487260; doi:10.1038/s44185-024-00063-5)

Supplementary Information

pH Adaptation Stabilizes Microbial Communities

Akihiko Mougi

SI text (Appendix):

Consider a case where two competing species (sp.1 and 2 are assumed to be acidophilic and alkaliphilic species, respectively) are not evolvable and have the physiologically optimal trait values ($p_i = \bar{p}_i$). For analytical tractability, I consider that the pH dynamics are very fast and at a quasi-equilibrium ($dY/dt = 0$). This is reasonable because the pH changes are caused by faster metabolism compared to the population dynamics. In this situation, the system is defined by the following differential equations:

$$dX_1/dt = (r_1 - X_1 - \alpha_{12}X_2)X_1, \quad (1)$$

$$dX_2/dt = (r_2 - X_2 - \alpha_{21}X_1)X_2, \quad (2)$$

where $Y = K_2X_2 - K_1X_1$ ($K_i = k_i/m$ is a normalized parameter by m). Then, I can show the equilibrium population sizes implicitly:

$$X_i^* = (r_i - r_j\alpha_{ij})/(1 - \alpha_{ij}\alpha_{ji}). \quad (3)$$

I conduct a local stability analysis of the equilibrium (3). The local stability of the system described by Eq. (1, 2) is analyzed by linearizing the dynamics near the nontrivial equilibrium. We can judge the stability by whether the characteristic equation of the Jacobian matrix satisfies the Routh-Hurwitz criteria. Under the equilibrium condition, I obtain the Jacobian matrix:

$$J = \begin{pmatrix} -X_1^*\left(1 - \left.\frac{\partial r_1}{\partial X_1}\right|_{X_i^*}\right) & X_2^*\left(\left.\frac{\partial r_2}{\partial X_1}\right|_{X_i^*} - \alpha_{21}\right) \\ X_1^*\left(\left.\frac{\partial r_1}{\partial X_2}\right|_{X_i^*} - \alpha_{12}\right) & -X_2^*\left(1 - \left.\frac{\partial r_2}{\partial X_2}\right|_{X_i^*}\right) \end{pmatrix}, \quad (4)$$

The characteristic equation for determining the eigenvalues is $\lambda^2 + \omega_1\lambda^1 + \omega_2 = 0$, where $\omega_1 = X_1^* (1 - \frac{\partial r_1}{\partial X_1}) + X_2^* (1 - \frac{\partial r_2}{\partial X_2})$ and $\omega_2 = X_1^*X_2^*\{1 - \alpha_{12}\alpha_{21} - \frac{\partial r_1}{\partial X_1}(1 - \frac{\partial r_2}{\partial X_2}) - \frac{\partial r_2}{\partial X_2} + \alpha_{21}\frac{\partial r_1}{\partial X_2} - \frac{\partial r_2}{\partial X_1}(\frac{\partial r_1}{\partial X_2} - \alpha_{12})\}$. The equilibrium is locally stable if $\omega_1, \omega_2 > 0$. Substituting $\frac{\partial r_i}{\partial X_i}$, the first condition ($\omega_1 > 0$) becomes:

$$\bar{X}^*/\theta > r_2K_2X_2^*(p_2 - Y) - r_1K_1X_1^*(p_1 - Y), \quad (5)$$

where $\bar{X}^*$ $(= (X_1^* + X_2^*)/2)$ is the mean equilibrium population size. The condition (5) is met if the r.h.s. of (5) is negative. The sufficient condition of (5) is the followings:

case I) $p_i < Y$, then it requires:

$$r_2K_2X_2^*/r_1K_1X_1^* > (p_1 - Y)/(p_2 - Y). \quad (6)$$

The condition (6) is likely to be met when $p_1 < 0 < p_2$ and $K_2X_2^* > K_1X_1^*$ $(Y > 0)$. The condition, $p_1 < 0 < p_2 < Y$, implies that in alkaline environment (produced by more productive alkaliphilic species than acidophilic species), each species is necessary to prefer the pH environment produced by themselves.

case II) $p_i > Y$, then it requires:

$$r_2K_2X_2^*/r_1K_1X_1^* < (p_1 - Y)/(p_2 - Y) \quad (7)$$

The condition (7) is likely to be met when $p_2 < 0 < p_1$ and $K_2X_2^* < K_1X_1^*$ $(Y < 0)$. The condition, $Y < p_2 < 0 < p_1$, implies that in acid environment (produced by more productive acidophilic species than alkaliphilic species), each species is necessary to prefer the pH environment produced by other species.

case III) $p_2 < Y < p_1$, then the condition (5) is always met. This implies that it requires a tendency that each species prefers a pH environment different from the one they themselves create, and an intermediate pH level.

Substituting $\frac{\partial r_i}{\partial X_i}$ and $\frac{\partial r_i}{\partial X_j}$, the second condition ($\omega_2 > 0$) is:

$$(1 - \alpha_{12}\alpha_{21})/2\theta > r_2(p_2 - Y)(K_2 + K_1\alpha_{12}) - r_1(p_1 - Y)(K_1 + K_2\alpha_{21}) \quad (8)$$

The sufficient condition of (8) is

$$1 > \alpha_{12}\alpha_{21} \quad (9)$$

and

$$r_2(p_2 - Y)(K_2 + K_1\alpha_{12}) < r_1(p_1 - Y)(K_1 + K_2\alpha_{21}) \quad (10)$$

If $p_1 > Y > 0 > p_2$ or $p_1 > 0 > Y > p_2$, the condition (10) is always met. Thus, it corresponds to the case III in first condition ($p_1 > Y > p_2$). Taken together, the sufficient condition for stability is reduced to $1 > \alpha_{12}\alpha_{21}$ and $p_1 > Y > p_2$. This implies that weaker interspecific competition than intraspecific competition, the opposite pH preferences from pH produced by themselves and an intermediate pH level are likely to stabilize the system.

The sufficient stability condition supports some result in the text. The stable communities are composed of species with the opposite pH preferences from pH produced by themselves (opposite pH preferences). Because of about half species with such opposite pH preferences in Fig. 4a, pH value is balanced in an intermediate level. This balance mechanism is also applied to the evolving communities. In the evolutionary stable states, the communities are comprised of about half species with the opposite pH preferences (Fig. S4a, S4c, S5a, S5c). The other patterns are also supported by the stability conditions: (i) lower pH sensitivity θ is likely to stabilize the system (Fig. 2a); (ii) higher pH self-regulation and/or lower pH change rate which makes K_i lower can approach the pH value to an intermediate level (Fig. S7a and S7c); and (iii) weaker interspecific competition stabilizes the system (Fig. S7b).

Supplemental Figures

Figure S1. Effects of pH sensitivity and niche width on community persistence. (a) $G = 0$. (b) $G = 0.5$. Contours represent the values of community persistence. Parameter values are $N = 30$, $C = 0.2$, $q = 0.6$, $m = 0.02$ and $\gamma = 2$.

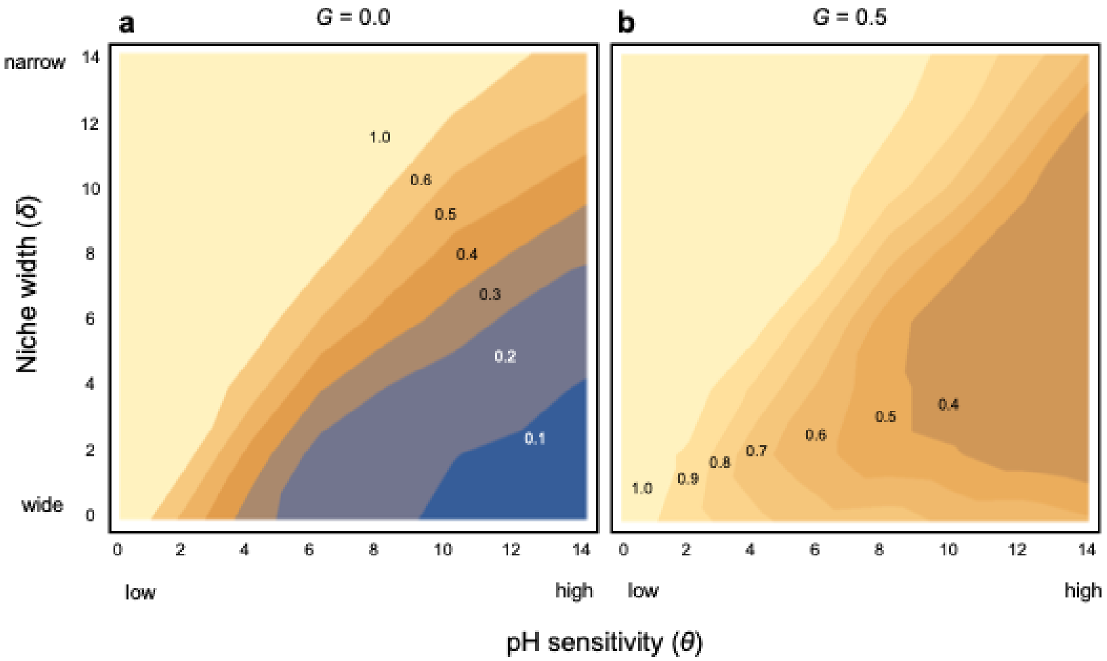

Figure S2. Effects of community composition on the equilibrium pH value. (a–c) q = 0.52. (d) q = 0.55. (e) q = 0.6. (f) q = 0.65. (a–c) has different parameter sets of pH sensitivity θ and cost strength γ, as shown in the upper side of each panel. (d–f) has the same parameter set of θ and γ, as shown in the upper side of each panel. Gray (and dark orange) bars are histograms of equilibrium pH value. Orange bars represent histograms of equilibrium pH value in only persistent communities. Parameter values are N = 30, C = 0.2, m = 0.02, δ = 15, and G = 0.5.

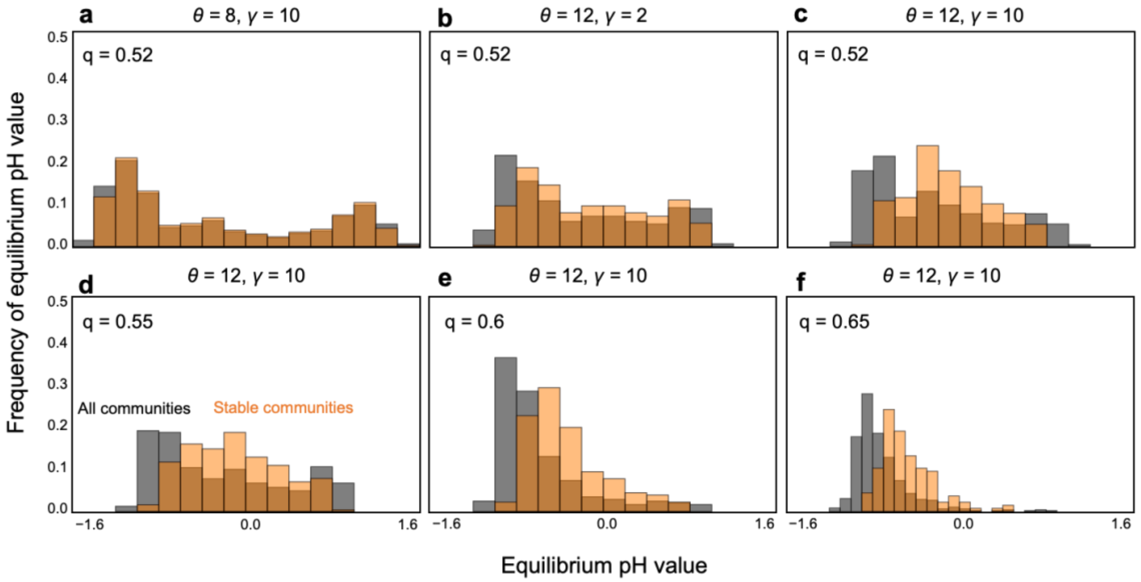

Figure S3. Relationship between community persistence and the distribution of equilibrium pH niches of communities. Different colors correspond to different speeds of adaptation (G = 0.05 (blue), 0.1 (red), 0.2 (yellow), 0.5 (green)). Standard deviation is used to calculate the distribution of equilibrium pH niches. Parameter values are N = 30, C = 0.2, q = 0.6, m = 0.02, θ = 8, δ = 15, and γ = 2.

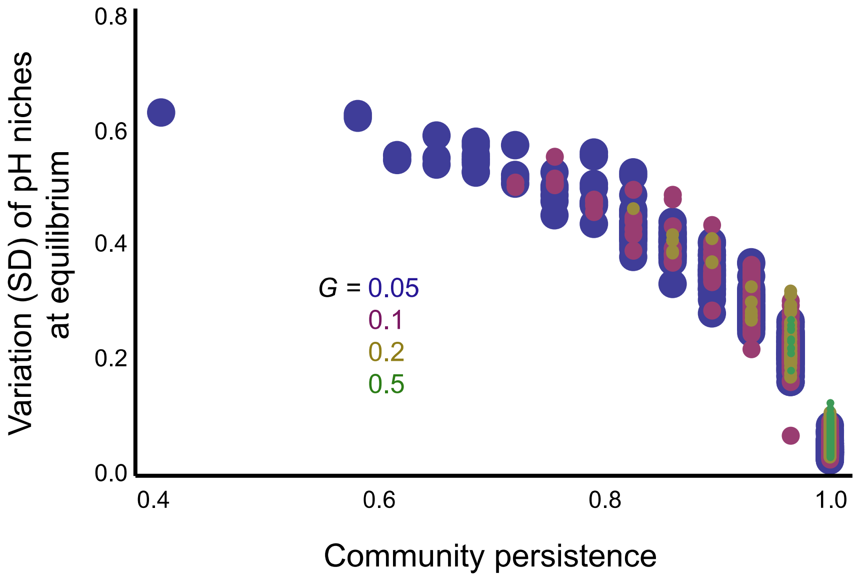

Figure S4. Match percentage of preference and change direction in pH at equilibrium. (a, c) q = 0.5. (b, d) q = 0.6. (a, b) G = 0.05. (c, d) G = 0.5. Gray (and darker orange) bars are the histograms of the match percentage of preference and change direction in pH at equilibrium. The match percentage of the signs of pH niche in each species at equilibrium and the signs of the direction of pH change by themselves were calculated during every simulation runs. Orange bars represent the histogram limited to the persistent communities. Parameter values are N = 30, C = 0.2, m = 0.02, θ = 8, δ = 15, and γ = 2.

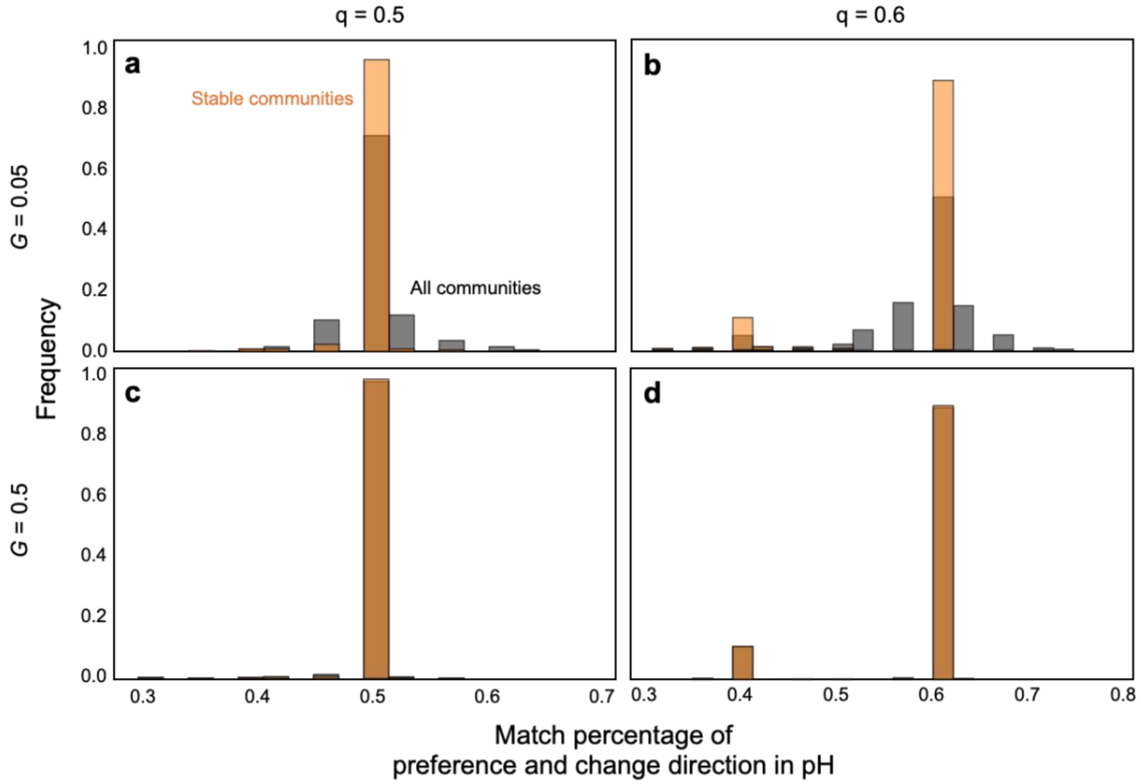

Figure S5. Distribution of pH niches at equilibrium. (a, c) q = 0.5. (b, d) q = 0.6. (a, b) G = 0.05. (c, d) G = 0.5. Gray (and dark orange) bars represent histograms of the proportion of species that prefers acid environment (pH niche is minus) at equilibrium. Orange bars represent histogram limited to the persistent communities. Parameter values are N = 30, C = 0.2, m = 0.02, θ = 8, δ = 15, and γ = 2.

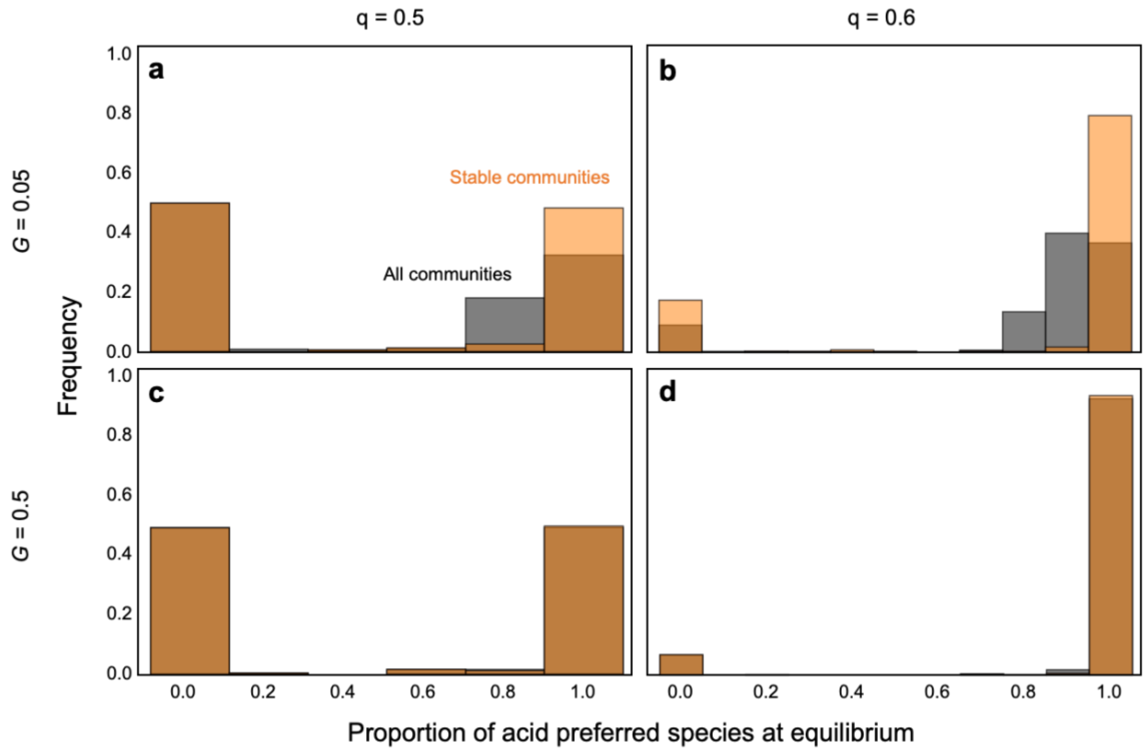

Figure S6. Relationship of pH niches between persistent and extinct species. (a) q = 0.5. (b) q = 0.6. Different colors correspond to different speeds of adaptation (G = 0.05 (blue), 0.1 (red), 0.2 (yellow), 0.5 (green)). The mean value of pH preferences at equilibrium was calculated for each of the extinct and survived species, during every simulation (the cases in which all species persisted were not included). Parameter values are N = 30, C = 0.2, m = 0.02, θ = 8, δ = 15, and γ = 2.

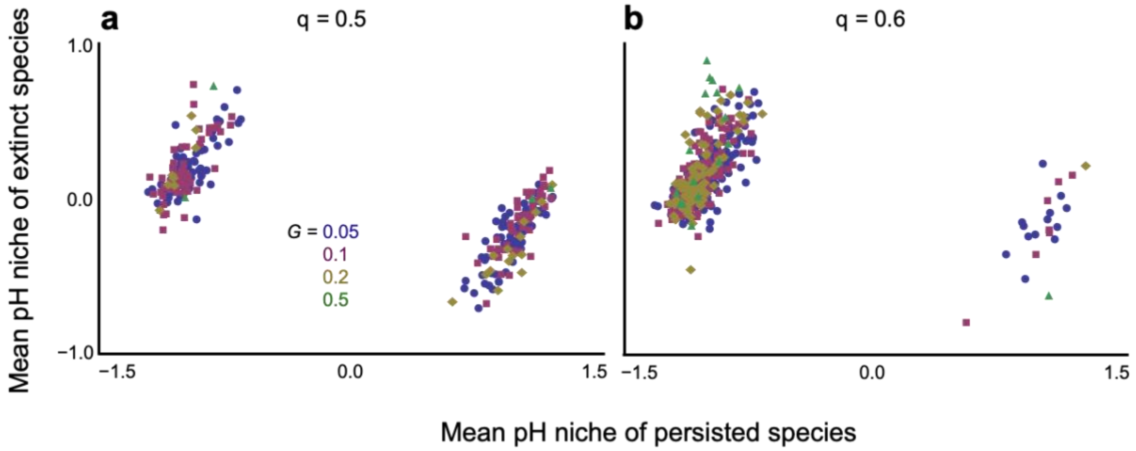

Figure S7. Parameter dependence of the effects of adaptation speed on community persistence. (a) Effect of pH self-regulation. α_{max} = 0.1, which is the maximum value of α_{0ij} (default value), and $|\boldsymbol{k_i}|_{\mathrm{max}}$ = 0.1, which is the maximum value of pH change rate (default value). (b) Effect of maximum strength of interspecific competition. m = 0.02 and $|\boldsymbol{k_i}|_{\mathrm{max}}$ = 0.1. (c) Effect of maximum pH change rate. α_{max} = 0.1 and m = 0.02. Different colors correspond to different speed of adaptation (G = 0 (blue), 0.1 (red), 0.2 (yellow), 0.5 (green)). Parameter values are N = 30, C = 0.2, q = 0.6, θ = 8, δ = 15, and γ = 2.

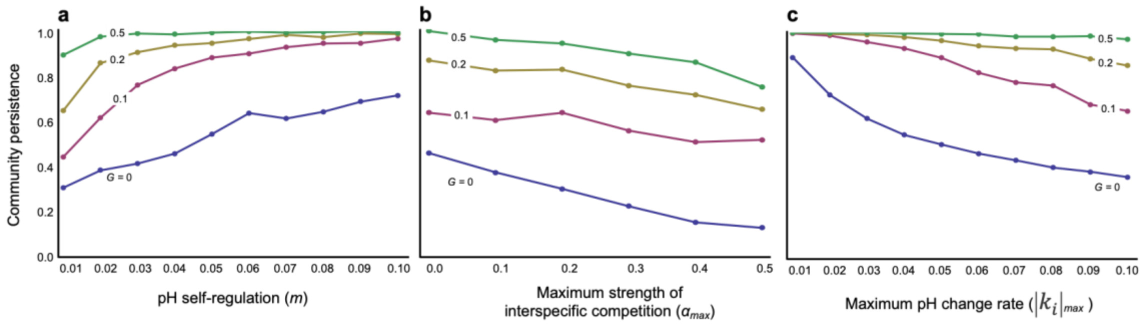

Figure S8. Effect of the community distribution of optimal pH values on community persistence. The standard deviation of the optimal pH distribution (σ) was varied (default value is 0.2 (see Material and Methods). Different colors correspond to different speed of adaptation (*G* = 0 (blue), 0.1 (red), 0.2 (yellow), 0.5 (green)). Parameter values are *N* = 30, *C* = 0.2, *q* = 0.6, *m* = 0.02, *θ* = 8, *δ* = 15, and *γ* = 2.

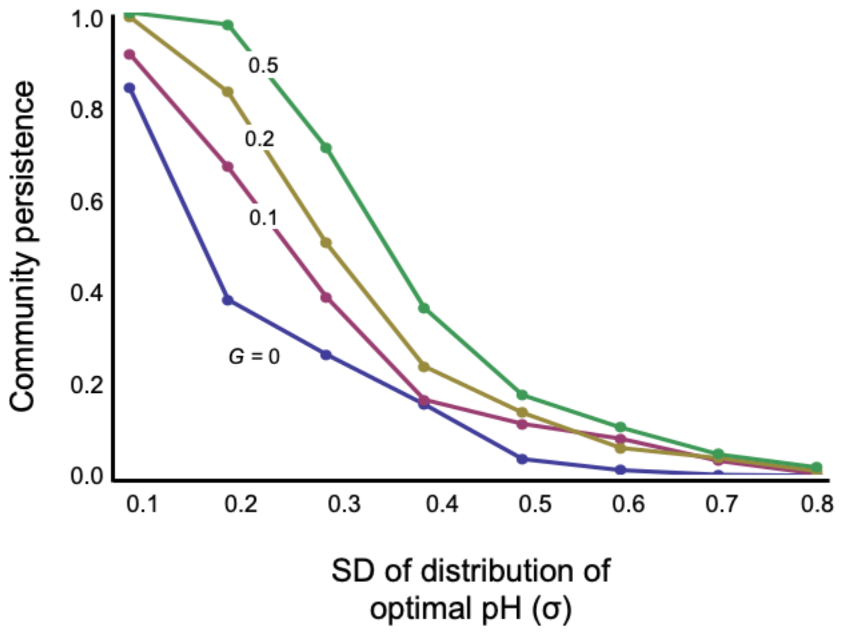

Figure S9. Relationship of characteristics of persistent and extinct species. (a) Relationship of mean growth rate between persisted and extinct species. (b) Relationship of mean optimal pH value between persisted and extinct species. Each dot represents the set of mean values of each parameter in persisted and extinct species, which were calculated in every simulations (the cases in which all species persisted are not included). Different colors correspond to different speed of adaptation (G = 0.05 (blue), 0.1 (red), 0.2 (yellow), 0.5 (green)). Parameter values are N = 30, C = 0.2, q = 0.6, m = 0.02, θ = 8, δ = 15, and γ = 2.

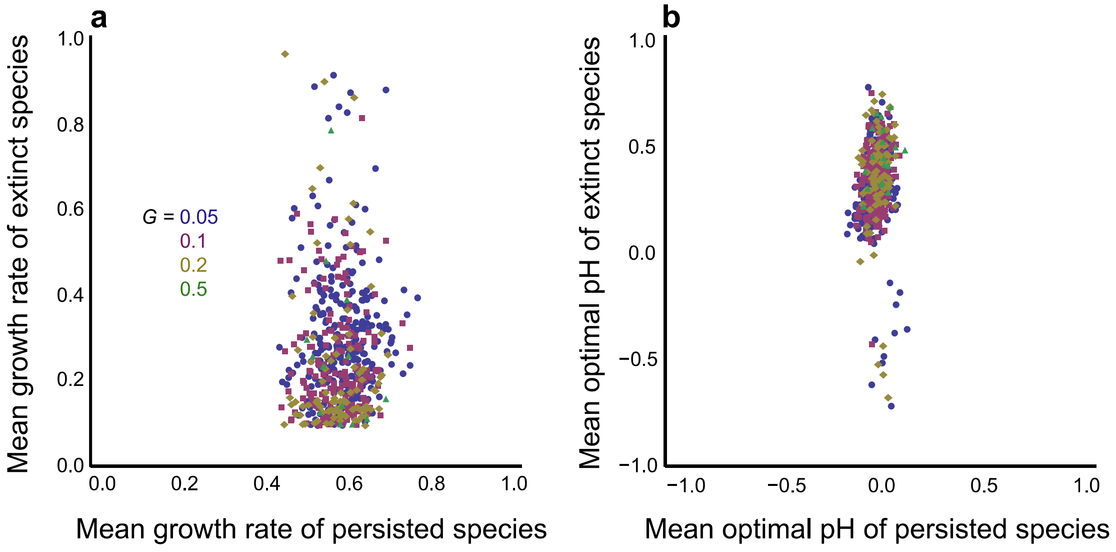

Supplement: Supplementary file 1 — Supplementary Information [file 44185_2024_63_MOESM1_ESM.pdf]
